# Delayed Diagnosis of Spinal Tuberculosis Presenting as Culture-Negative and Biopsy-Negative Vertebral Osteomyelitis: A Case Report

**DOI:** 10.51894/001c.165693

**Published:** 2026-07-31

**Authors:** Joshua Marwede, Sai Medavaka, Srimukhi Karanam

**Affiliations:** 1 Henry Ford - Macomb

## 99

### Introduction

Spinal tuberculosis is the most common form of skeletal tuberculosis but often presents with nonspecific symptoms such as chronic back pain. Because early clinical findings resemble other causes of vertebral infection, particularly vertebral osteomyelitis, diagnosis may be significantly delayed. Conventional diagnostic methods may remain inconclusive in extrapulmonary tuberculosis. As a result, patients are often treated empirically for bacterial osteomyelitis before the underlying diagnosis is established. The disease typically develops insidiously, and early symptoms may be mistaken for mechanical injury or degenerative spinal disease. Advances in molecular diagnostics, including polymerase chain reaction (PCR) testing, have improved detection of Mycobacterium tuberculosis in extrapulmonary infections and are particularly useful when routine cultures are negative.

### Case Description

A 60 year-old woman presented with persistent thoracolumbar back pain initially attributed to mechanical injury following a workplace incident in 2023 and a subsequent motor vehicle accident. Despite conservative therapy and spinal steroid injections, her pain progressively worsened. Magnetic resonance imaging demonstrated findings concerning for discitis and osteomyelitis localized to the T12 vertebral body. Due to progressive symptoms and vertebral destruction, the patient ultimately underwent T12 corpectomy with thoracolumbar fusion from T10 to L3 with laminectomy. Intraoperative biopsies and blood cultures remained negative. Postoperative molecular testing of vertebral bone tissue using PCR confirmed Mycobacterium tuberculosis, establishing the diagnosis of spinal tuberculosis. The patient was started on standard antituberculous therapy. Evaluation for pulmonary tuberculosis with sputum PCR and acid-fast bacilli testing was negative, suggesting infection localized to the spine.

### Discussion

Spinal tuberculosis can closely mimic other causes of vertebral osteomyelitis, making diagnosis particularly challenging when traditional cultures and biopsies are negative. This case highlights the limitations of conventional diagnostic approaches and underscores the value of molecular techniques such as PCR in identifying Mycobacterium tuberculosis in culture-negative infections. Delayed diagnosis may lead to progressive vertebral destruction and structural instability requiring surgical intervention. Clinicians should maintain a high index of suspicion for spinal tuberculosis in patients with persistent vertebral infection despite negative cultures, particularly when epidemiologic risk factors are present. Early recognition and the use of molecular diagnostic testing are essential to prevent delays in treatment.

